# Bacterial Cellulose: A Sustainable Source for Hydrogels and 3D-Printed Scaffolds for Tissue Engineering

**DOI:** 10.3390/gels10060387

**Published:** 2024-06-05

**Authors:** Elena Utoiu, Vasile Sorin Manoiu, Elena Iulia Oprita, Oana Craciunescu

**Affiliations:** National Institute of R&D for Biological Sciences, 296, Splaiul Independentei, 060031 Bucharest, Romania; elena.utoiu@incdsb.ro (E.U.); sorin.manoiu@incdsb.ro (V.S.M.); oana.craciunescu@incdsb.ro (O.C.)

**Keywords:** bacterial cellulose, hydrogel, ink, 3D printing, wound healing, tissue engineering

## Abstract

Bacterial cellulose is a biocompatible biomaterial with a unique macromolecular structure. Unlike plant-derived cellulose, bacterial cellulose is produced by certain bacteria, resulting in a sustainable material consisting of self-assembled nanostructured fibers with high crystallinity. Due to its purity, bacterial cellulose is appealing for biomedical applications and has raised increasing interest, particularly in the context of 3D printing for tissue engineering and regenerative medicine applications. Bacterial cellulose can serve as an excellent bioink in 3D printing, due to its biocompatibility, biodegradability, and ability to mimic the collagen fibrils from the extracellular matrix (ECM) of connective tissues. Its nanofibrillar structure provides a suitable scaffold for cell attachment, proliferation, and differentiation, crucial for tissue regeneration. Moreover, its mechanical strength and flexibility allow for the precise printing of complex tissue structures. Bacterial cellulose itself has no antimicrobial activity, but due to its ideal structure, it serves as matrix for other bioactive molecules, resulting in a hybrid product with antimicrobial properties, particularly advantageous in the management of chronic wounds healing process. Overall, this unique combination of properties makes bacterial cellulose a promising material for manufacturing hydrogels and 3D-printed scaffolds, advancing the field of tissue engineering and regenerative medicine.

## 1. Introduction

In recent years, bacterial cellulose has been a subject of interest for researchers due to its unique macromolecular structure and biological properties [1,2,3,4,5]. Early studies focused on optimizing the production and purification methods of bacterial cellulose, in order to improve its quality and suitability for various biomedical applications [6,7]. The material was predominantly utilized to design wound dressings, tissue engineering scaffolds, and controlled drug delivery systems [4,5]. However, its utilization in 3D printing for biomedical purposes was relatively limited due to challenges in processing and scalability [8]. Research efforts in the past have laid the foundation for understanding the fundamental characteristics of bacterial cellulose and exploring its potential in the realm of biomedicine [4,5,9].

Bacterial cellulose is a natural biopolymer produced by various species of bacteria, such as *Rhizobium*, *Gluconacetobacter*, *Sarcina*, *Komagataeibacter*, *Agrobacterium*, and *Rhodobacter* [10], and is known for its remarkable properties, including high purity, biocompatibility, mechanical strength, and water retention capacity [4]. These unique characteristics make bacterial cellulose an attractive material for a wide range of biomedical applications, with 3D printing emerging as a promising avenue for its utilization. Traditional biomaterials used in 3D printing, such as synthetic polymers, often face limitations in terms of biocompatibility, degradation rates, and mechanical properties. In contrast, bacterial cellulose offers a sustainable and eco-friendly alternative that has the potential to address these challenges and revolutionize the field of biomedical 3D printing.

The scientific community is starting to show an increased interest in harnessing the capabilities of bacterial cellulose for 3D printing applications in the biomedical field [4,5,11]. Researchers are actively exploring innovative strategies to functionalize bacterial cellulose-based materials with bioactive compounds to enhance their performance in tissue regeneration, drug delivery, and medical device manufacturing [7,12]. Advances in additive manufacturing technologies have facilitated the development of customized 3D printing techniques and parameters that allow the fabrication of intricate structures using bacterial cellulose-based composites with improved mechanical properties and structural integrity [5,7,9]. Current research endeavors are focused on evaluating the biocompatibility, degradation kinetics, and efficacy of bacterial cellulose-based 3D printed constructs in both in vitro and in vivo settings across a wide spectrum of biomedical applications [9,13].

**Figure 1:**
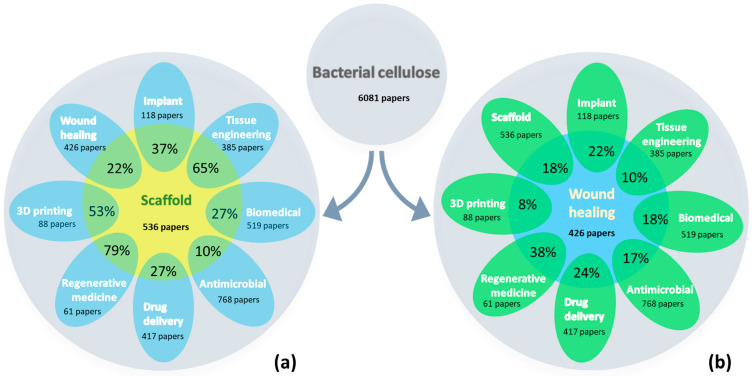
Correlation between publications on bacterial cellulose found as a common denominator in the Web of Science databases between 2020 and April 2024, and (**a**) scaffold and (**b**) wound healing, and distribution according to the most addressed applications.

The number of scientific papers regarding bacterial cellulose has significantly increased over the last 10 years, but the most rapid growth was observed in the last five years, specifically between 2020 and 2024, when over half of the publications were published. When searching for the term “bacterial cellulose”, 6081 publications in total were found in the online Web of Science core collection database in 2020–2024. An analysis of these publications on “bacterial cellulose”, as a common denominator, and “scaffold” or “wound healing”, indicated 536 and 426 articles, respectively, and revealed an uneven distribution according to the most addressed applications, as depicted in Figure 1. A high percentage was found when “regenerative medicine” was superimposed.

**Figure 2:**
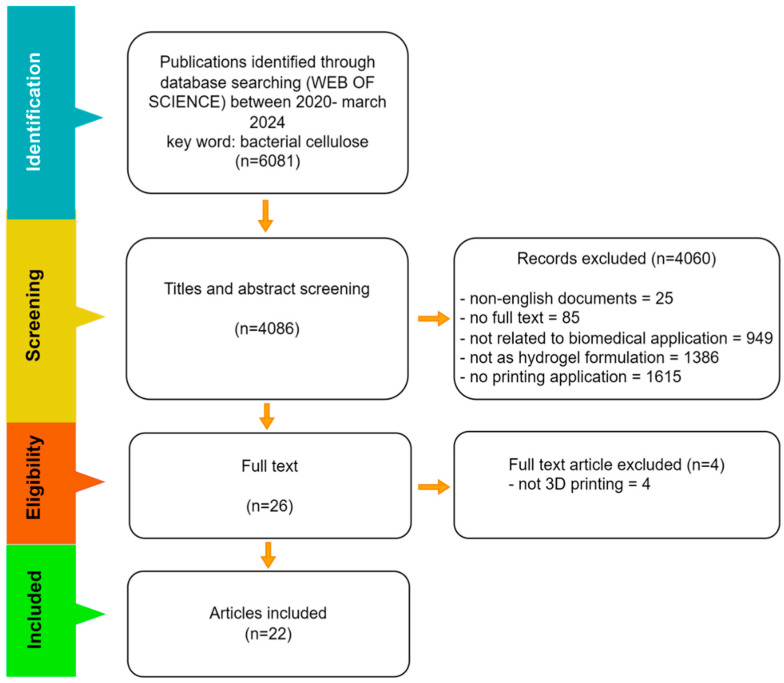
Flowchart of the articles found in the Web of Science database between 2020 and April 2024.

To refine this search, the search strategy included the keywords “bacterial cellulose”, “hydrogel”, and “3D printing”, and the research domain of biomedical applications, inclusive of “tissue engineering”, “drug delivery”, and “wound healing”. In total, 22 full-text articles fit all our inclusion criteria (Figure 2).

In this context, the present paper aims to explore the current landscape of promising research on bacterial cellulose, highlighting its physicochemical and structural properties, biocompatibility, formulation as hydrogel (ink), and its customizability for applications in the field of biomedicine with a specific focus on the emerging 3D printing technology.

## 2. Bacterial Cellulose Hydrogels—Physicochemical, Structural, and Biological Properties

Hydrogels play a vital role in various biomedical applications, providing a hydrated, biocompatible matrix for cell encapsulation, tissue regeneration, and controlled drug delivery. The numerous properties of hydrogels make them indispensable tools in tissue engineering, providing a versatile platform for creating biomimetic tissue constructs, promoting cell growth and tissue regeneration, allowing for controlled drug delivery, and facilitating advanced research and therapeutic applications in regenerative medicine [7]. Their biocompatibility, adjustable characteristics, and capacity to imitate the natural tissue milieu make them important in the advancement of tissue engineering and regenerative medicine. Certain hydrogels, for example, can alter tissue engineering by acting as biomimetic scaffolds that mimic the mechanical properties of genuine tissues, boosting integration with surrounding tissues and cell viability [14]. Studies have shown that by modifying the mechanical properties of hydrogels, it is possible to meet specific needs of tissue engineering applications for bone, cartilage, and blood vessels.

The main components used as building blocks for hydrogels in tissue engineering include natural polymers such as alginate, elastin, chitosan, silk fibroin, fibrinogen, collagen, hyaluronic acid, and gelatin, along with synthetic polymers like poly (ethylene glycol), methacrylates, polyvinyl alcohol, polyacrylate, and polyacrylamide [9,15,16]. However, most of the time, these materials provide limited mechanical properties or may not contain nano-to-mesoscale topographic features. As a result, hydrogels based on self-assembling/fibrillizing (bio)molecules or nanofibrils, such as bacterial cellulose, are appealing due to their ease of chemical modification, advanced and tunable mechanical properties, and the potential for the mesoscale alignment of colloidal scale nanofibrils to guide cell alignment [15,17]. The presence of many hydroxyl groups on the surface of the cellulose matrix allows for functionalization while maintaining the surface’s hydrophobicity and thermodynamic stability [9,18].

### 2.1. Bacterial vs. Plant-Derived Cellulose

**Figure 3:**
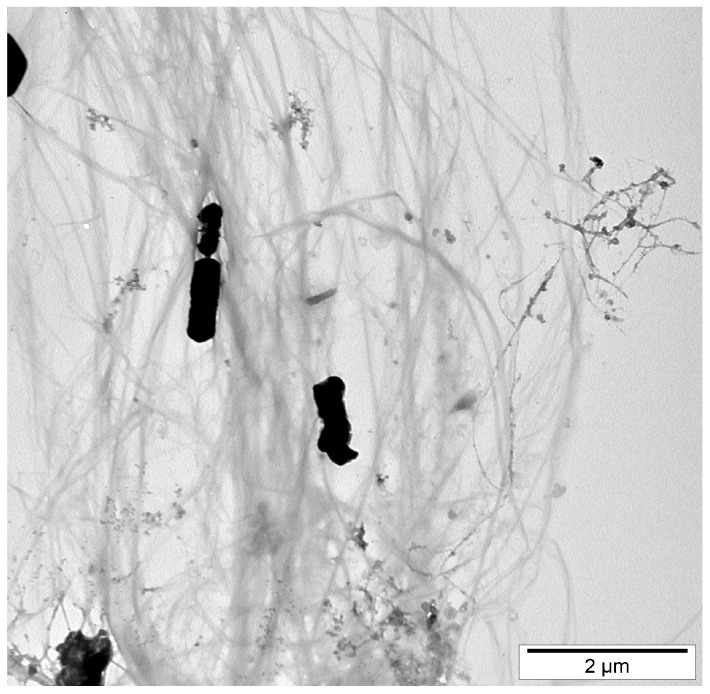
Transmission electron micrograph of bacterial nanocellulose obtained via fermentation of *Equisetum arvense* in Kombucha consortia of yeasts and bacteria (unpublished results obtained by the authors).

Cellulose, a polysaccharide comprising β-1,4 connected D-glucose units, is one of the most prevalent biodegradable materials on the planet. The main source of cellulose is represented by plants and agricultural residues, including wood, cotton, hemp, bamboo, and sisal bagasse. On the other hand, cellulose can be generated by a number of microorganisms from the genera *Acetobacter*, *Gluconobacter*, *Komagataeibacter*, *Rhizobium*, *Agrobacterium*, and *Sarcina* [10]. Bacterial cellulose is also manufactured by specific lactic and acetic acid bacteria in SCOBY consortia in a regulated fermentation process, yielding a very pure and consistent form of cellulose nanofibrils [19]. Therefore, bacterial cellulose’s purity and uniformity make it more suitable for scientific and industrial applications that require careful control over material qualities. Unlike plant-derived cellulose, which is a primary component of plant cell walls, bacteria generate cellulose as a mechanism to protect themselves and establish a stable environment for growth (Figure 3).

Although both types of cellulose share some similar characteristics, bacterial cellulose is often preferred over plant cellulose as a base material in hydrogel composition for several reasons: its purity and consistency, nanostructure, mechanical properties, biocompatibility, low thermal expansion, high optical transparency, scalability, and efficiency [20] (Table 1).

**Figure 4:**
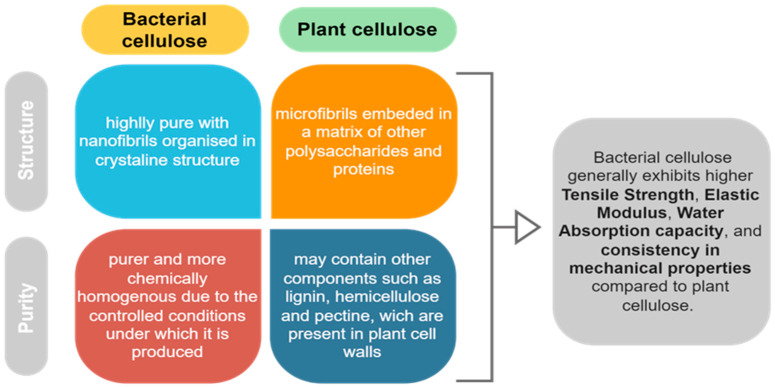
Main differences between structure and purity of bacterial and plant cellulose.

Bacterial cellulose has a distinct nanostructure that includes a highly porous network of nanofibers. Cellulose chains form basic fibrillary units or elementary fibrils with lengths of 0.1 to 0.2 μm organized into microfibrils measuring 0.1 μm in width and 0.1 to 1 μm in length [21]. This structure improves water absorption and retention, these being preferred for hydrogel production, which requires high water content (Figure 4).

In regard of mechanical properties, bacterial cellulose has a significant tensile strength (200 MPa) [22], while plant-derived cellulose has a value of tensile strength of up to 1 GPa. Still, bacterial cellulose presents higher flexibility with a Young modulus of 15 GPa vs. plant-derived cellulose with a Young modulus of 130 GPa. These mechanical qualities make it ideal for applications requiring strength and durability, such as wound dressings and other biomedical devices. Due to its purity, bacterial cellulose showed excellent biocompatibility, and is widely adopted as a scaffolding biomaterial for the modeling and engineering of biomimetic tissues, showing appropriate cellular response and behavior [23].

Another aspect to consider is the environmental impact of the chosen cellulose source. While vegetal cellulose is sourced from renewable plant materials, bacterial cellulose manufacturing can be more efficient and scalable, particularly when using specific bacterial strains in controlled fermentation conditions. Because of its high efficiency, bacterial cellulose may be a more cost-effective solution for the manufacture of large-scale hydrogel and other materials [24].

### 2.2. Properties of Bacterial Cellulose

Bacterial cellulose is characterized by a highly pure (i.e., not containing hemicellulose, lignin, pectin, or wax) and uniform structure composed of interconnected cellulose nanofibers. It possesses distinct features including good mechanical strength, high crystallinity (80–90%), ultrafine nanofibrils forming networked bulk structures [25,26,27], high water holding capacity [28], and excellent biocompatibility [23].

In recent years, bacterial cellulose has gained increasing attention as a base material for hydrogel formulation, particularly in the context of 3D printing. Its unique architecture imparts exceptional mechanical strength and structural integrity to hydrogels, making them well suited for 3D printing applications that demand precise control over shape, resolution, and design complexity [29]. Better mechanical performance, even in wet conditions, was achieved via the ion crosslinking of bacterial cellulose nanofibers using Fe^3+^ to obtain macrofibers with higher tensile strength [30]. The nanofibrous network of bacterial cellulose offers a biomimetic scaffold that mimics the microstructural, physicochemical, and mechanical characteristics of the extracellular matrix, promoting cell adhesion, proliferation, and differentiation, and favoring oxygen and nutrient permeability [31]. The natural constituents also provide biophysical and biological cues to stimulate and direct cell function and signaling through cell surface receptors, growth factors, or signal proteins [32]. Microscopically, bacterial nanocellulose fibrils resemble collagen fibrils, presenting the same size range, i.e., a width of roughly 100 nm [33,34,35].

Bacterial cellulose hydrogels possess a remarkable capacity for water absorption and retention, creating a hydrated microenvironment conducive to cell viability, proliferation, and differentiation. The ability of bacterial cellulose to retain water molecules within its porous structure enhances nutrient transport, waste removal, and signaling molecule diffusion, essential for supporting cellular activities within the hydrogel matrix [33,36].

The thermal characteristics of hydrogels are closely related to their viscosity and therefore to their printability, and thermographic analysis provides important data to determine the parameters, compositions, and properties of hydrogel viscosity [36,37]. Rheological investigations showed the flow properties of hydrogels used for the 3D printing of scaffolds, viscosity being an essential parameter in determining printability. Also, rheological experiments were conducted to evaluate the changes in the mechanical properties of bacterial cellulose hydrogel that occurred during the UV curing process [38]. The linear and non-linear tests provided information on temperature, component, and water concentration that can greatly influence the thermoelastic properties and behavior of hydrogels when printed, and the subsequent mechanical properties of 3D-printed structures [36,39,40,41,42,43,44].

A key advantage of bacterial cellulose is its biocompatibility, making it an attractive choice for biomedical applications where interaction with living tissues is paramount. Hydrogels incorporating bacterial cellulose exhibit minimal cytotoxicity and immunogenicity, enabling their safe use in tissue engineering, wound healing, and regenerative medicine [45]. Recently, an improvement in cell adhesion and a growth of cells were reported after the surface modification of bacterial cellulose via drying and plasma treatment to obtain higher porosity and hydrophylicity [46]. Moreover, the biodegradability of bacterial cellulose ensures that the hydrogel scaffold can gradually degrade over time, allowing for tissue ingrowth and remodeling.

**Figure 5:**
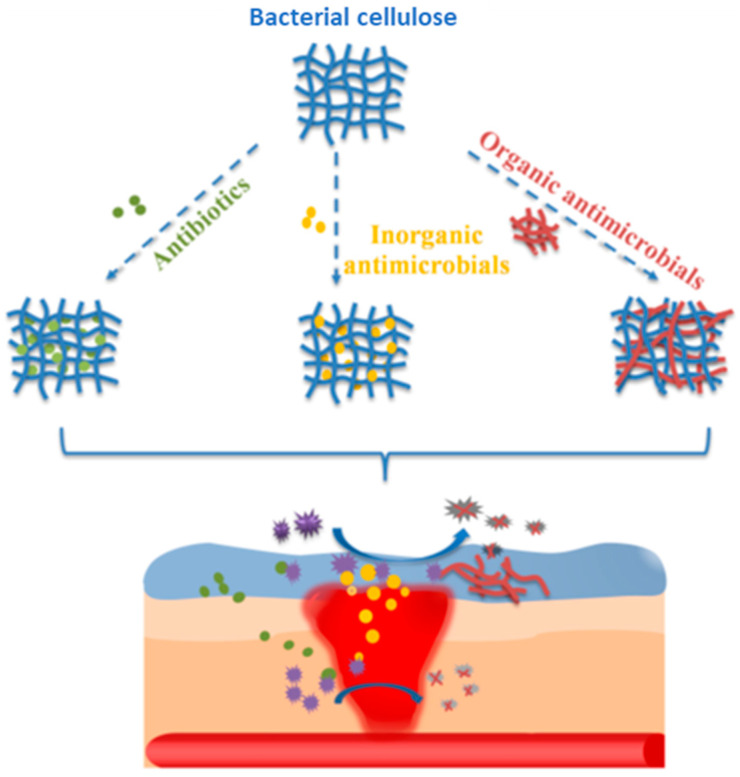
Bacterial cellulose-based antimicrobial biomedical device for skin wound healing. Reprinted from [47] under a Creative Commons Attribution License 4.0 (CC BY), “CC BY 4.0 Deed|Attribution 4.0 International|Creative Commons (accessed on 21 May 2024)”.

Bacterial cellulose itself has no antimicrobial activity, but due to its ideal structure (including an ultrafine and highly porous network of nanofibrils), it can serve as a matrix for other biologically active molecules (Figure 5). Thus, it becomes an essential part of hybrid products with antimicrobial applications [47] that provide a large contact surface area for interactions with microorganisms [48].

Nanocellulose-based antimicrobial materials are classified into two types based on the used antimicrobial agent: inorganic (silver nanoparticles) [48,49,50,51,52,53] and organic (honey, curcumin, antibiotics) [54,55,56]. Acting as a stabilizer of the antimicrobial formulation, bacterial cellulose can sustain controlled release and improve the bioactivity of poorly soluble compounds [48]. Therefore, more effectiveness in the targeted biomedical field can be provided via the structural and physical modification of bacterial cellulose, on the one hand, and the process of in situ or ex situ tuning using antimicrobial compounds or polymers, on the other hand [57].

In regenerative medicine, bacterial cellulose promotes cellular adhesion, proliferation, migration, and differentiation to accelerate re-epithelization and speed up wound healing [10,27,45,58]. The molecular mechanisms revealed during these processes were based on the interleukin 10 (IL 10) signaling pathway, with important immunoregulatory function and vascular endothelial growth factor (VEGF) signaling involved in cell proliferation and angiogenesis [10]. Furthermore, bacterial cellulose has wide-ranging uses in biomedical device manufacturing, including dental implants, artificial blood vessels, vascular grafts, implants for the urethra and nerve, artificial corneas, and retinas. Its main characteristics are low tissue adhesion, low toxicity, thermal insulation, and preservation of a moist environment for gas exchange at the wound site [10,24,45,59,60,61]. For bacterial cellulose to function in vivo, cell–cell contact is essential [4,5,9,10,11,12,13,14,15,16,27]. More importantly, because of its topography, wettability, and surface charge, bacterial cellulose demonstrates remarkable physicochemical and pharmacological properties [1,4,27,59,60]. In tissue engineering applications, bacterial cellulose is used in two main ways: as grown directly on application-specific substrates, or mechanically and chemically processed to serve as a reinforcing or crosslinking agent. The crystallinity of bacterial cellulose is the main property correlated to its reinforcing ability [61].

## 3. Applications of Bacterial Cellulose Hydrogels in 3D Printing Technologies

### 3.1. Customizability and Tunability for 3D Printing

**Figure 6:**
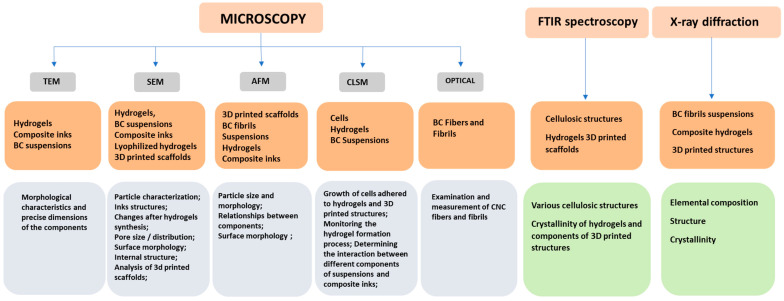
Main techniques used for the morphological and structural characterization of bacterial cellulose-based hydrogels and 3D-printed scaffolds. BC—bacterial cellulose.

The mechanical properties, porosity, degradation rate, and bioactive functionalities of bacterial cellulose-based hydrogels can be tailored through various processing techniques, such as blending with polymers, the incorporation of bioactive molecules, or physical crosslinking methods. This customizability allows researchers to fine-tune the properties of the hydrogel to meet the specific requirements of diverse applications, ranging from soft tissue engineering to cartilage regeneration and drug delivery systems. Various investigation techniques can be used to assess and optimize hydrogel properties (Figure 6).

Thus, the micromorphology, fibril length, and thickness of unmodified or modified bacterial cellulose diluted solutions were investigated via transmission electron microscopy (TEM) [45,61], this being essential for determining the tunability of hydrogels in view of 3D printing. Also, a modification on the surface of bacterial-generated cellulose nanofibers with 3-(trimethoxysilyl)propyl methacrylate (TMSPM) was revealed via TEM at an accelerating voltage of 200 kV [61].

**Figure 7:**
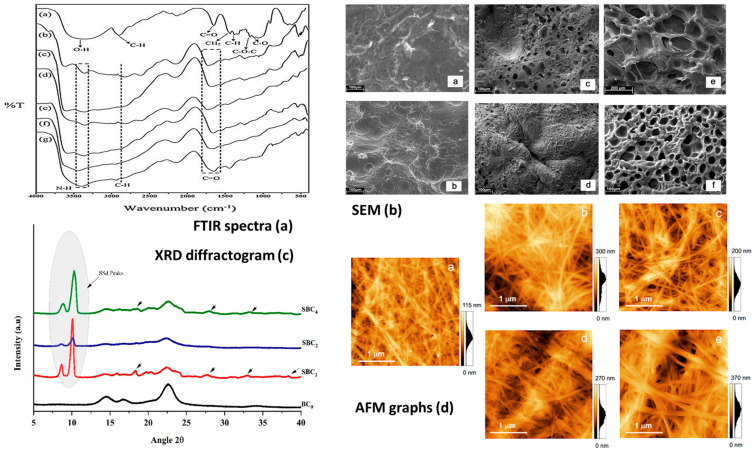
(**a**) FT-IR spectra of bacterial cellulose–gelatin composite hydrogels (a–g) at varying mixing ratios between 25:1 and 400:1, reprinted from [65], Copyright (2017), with permission from Elsevier, under a Creative Commons Attribution License 4.0 (CC BY), “CC BY 4.0 Deed|Attribution 4.0 International|Creative Commons (accessed on 28 May 2024)”; (**b**) SEM images of dense networks of bacterial cellulose–acrylic acid hydrogels (a–f) with improved thermal stability, reprinted from [63], Copyright (2012), with permission from Elsevier, under a Creative Commons Attribution License 4.0 (CC BY), “CC BY 4.0 Deed|Attribution 4.0 International|Creative Commons (accessed on 28 May 2024)”; (**c**) X-ray diffraction (XRD) patterns of bacterial cellulose–chitosan hydrogels indicating major peaks (arrow) corresponding to crystalline structures after the addition of silver sulfadiazine, reprinted from [67], Copyright (2021), with permission from Elsevier, under a Creative Commons Attribution License 4.0 (CC BY), “CC BY 4.0 Deed|Attribution 4.0 International|Creative Commons (accessed on 28 May 2024)”; (**d**) AFM graphs indicating thicker nanofibers of bacterial cellulose and the reinforcement of polymerizable choline chloride–acrylic acid mixtures (a–e) for 3D printing, reprinted from [62] under a Creative Commons Attribution License 4.0 (CC BY), “CC BY 4.0 Deed|Attribution 4.0 International|Creative Commons (accessed on 28 May 2024).

An atomic force microscopy (AFM) study demonstrated a thickening of bacterial cellulose nanofibers from 55–95 nm to 85–140 nm via poly(acrylic acid) grafting on their surface during UV-induced polymerization, which reinforced the mixtures for the 3D printing of stable structures (Figure 7) [62].

SEM investigations showed modifications to surface morphology and pore size variation between 2 and 190 µm in bacterial cellulose hydrogels, according to the increasing content of acrylic acid in their composition, presenting denser inter-polymeric networks after electron beam irradiation and improved thermal stability (Figure 7) [63]. An investigation of the in situ arrangement of bacterial cellulose filaments in spheroids and of their size and shape was conducted via optical, SEM, and TEM analyses to allow for a tunable strategy for biomedical applications [64].

Changes in the functional groups of the hydrogels components were highlighted via Fourier transform infrared spectrometry (FT-IR) to determine the optimal chemical structure of bacterial cellulose hydrogels used as inks for 3D printing [10]. Improvements in mechanical properties and the thermal stability of bacterial cellulose–gelatin composite hydrogels at varying mixing ratios between 25:1 and 400:1 were observed via FT-IR, highlighting hydrogen bond formation between amine and hydroxyl groups (Figure 7) [65]. Alongside this, the structural characteristics and crystallinity of bacterial celluloses investigated via Raman spectroscopy and X-ray diffraction varied according to static or dynamic regimes of cultivation with *Gluconacetobacter sucrofermentans*, allowing the selection of parameters to strengthen the derived inks [66]. Crystalline regions alternating with amorphous ones were revealed after the addition of silver sulfadiazine to bacterial cellulose-chitosan hydrogels, via X-ray diffraction, simultaneously improving the mechanical and antibacterial properties required for tissue engineering applications (Figure 7) [67].

Changes to surface wettability observed via contact angle measurements in nanofibrous porous microstructures of bacterial cellulose revealed enhanced biocompatibility and the potential to interact with mouse embryonic stem cells [68]. In addition, optimal values of bacterial nanocellulose concentration and mixing ratios with gelatin methacryloyl influenced the mechanical properties of composite hydrogels intended for 3D printing [69].

The intrinsic properties of bacterial cellulose, including its structural integrity, biocompatibility, and water retention capabilities, make it highly compatible with 3D printing technologies. By integrating bacterial cellulose as a base element in hydrogel formulations for 3D printing, researchers can achieve precise control over the spatial distribution of cells, growth factors, and biomaterials, enabling the fabrication of complex, multi-material constructs with tailored mechanical and biological properties [9,12,70]. The versatility of 3D printing techniques, such as extrusion-based printing, stereolithography, and inkjet bioprinting, further enhances the potential of bacterial cellulose hydrogels for creating patient-specific implants, organ-on-a-chip models, and personalized medical devices.

By applying living cells, also known as bioink, to a non-living surface, living items can be 3D printed. The structural integrity of the entire 3D design and the survival and functionality of the cells in the bioprinted structures depend heavily on the characteristics of the printing substrate and the makeup of the bio-ink [71]. On the other hand, cell behavior, beyond the physical and chemical properties of the hydrogel, is also influenced by its 3D geometrical structure, since a better resemblance to the extracellular matrix leads to better cell proliferation. Therefore, for biomedical applications targeting the preparation and conditioning of nanocellulose-based hydrogels, a number of methods have been developed to generate a 3D structure that mimics the extracellular matrix, including homogenization, freeze–thawing cycling, freeze-drying, free radical polymerization, photocrosslinking and 3D bioprinting [13,72].

### 3.2. Applications of Bacterial Cellulose Hydrogels

Because of their biocompatibility, mechanical robustness, and bioactive properties, bacterial cellulose hydrogel applications include wound healing, drug delivery systems, and tissue engineering [11,12,73]. Synthesized data on recent advances are presented in Table 2.

### 3.3. Characterization of Bacterial Cellulose-Based 3D-Printed Scaffolds

**Figure 8:**
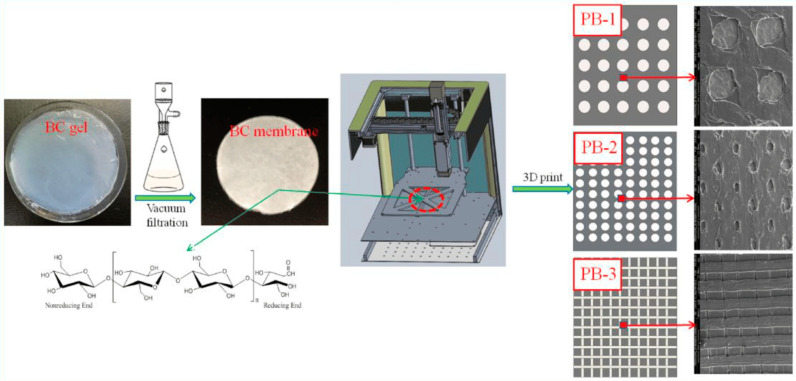
SEM images of composite scaffolds of polylactic acid/bacterial cellulose (PB-1, PB-2, and PB-3) prepared by 3D printing, showing different surface microstructures for tissue engineering applications. Reprinted from [82] under a Creative Commons Attribution License 4.0 (CC BY), “CC BY 4.0 Deed|Attribution 4.0 International|Creative Commons (accessed on 30 May 2024)”.

For the characterization and analysis of bacterial cellulose-based 3D-printed scaffolds, several investigation techniques serve to assess their morphological, structural, and mechanical properties and their biocompatibility (Figure 6). Various microscopy techniques, such as optical, transmission and scanning electron, atomic force, and confocal microscopy were used for the morphological and structural characterization of bacterial cellulose 3D-printed structures. Each microscopy technique has its own advantages and disadvantages, such as equipment resolution, the complexity of sample preparation, the time required, and last but not at least the cost of conducting a good evaluation of certain morphological characteristics of 3D-printed bacterial cellulose scaffolds. Thus, SEM of polylactic acid 3D-printed composite scaffolds containing bacterial cellulose facilitated stability evaluation via observations of strand width, with the morphology of the surface and pore size, and the microstructure controlled via layer printing (Figure 8) [82].

**Figure 9:**
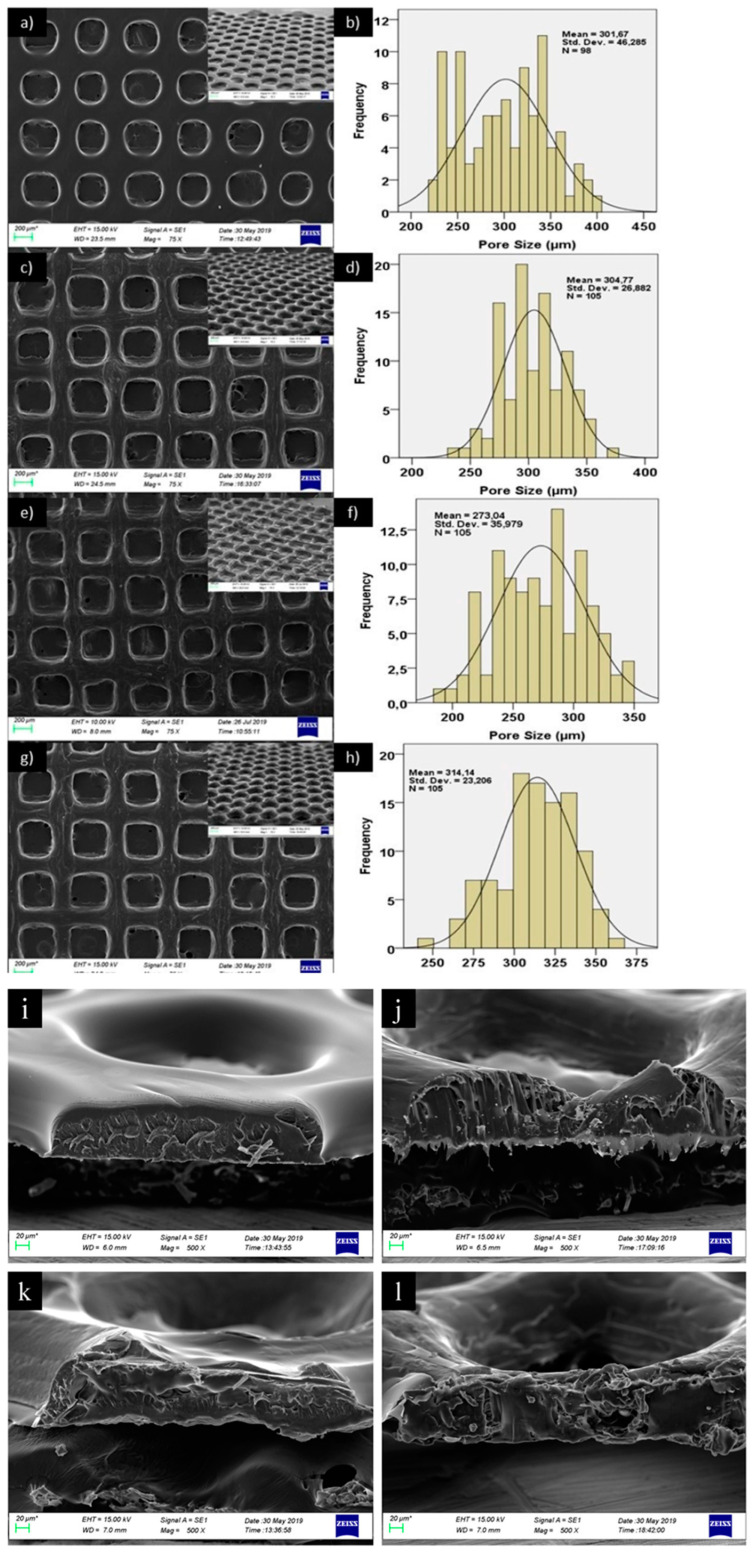
Pore shape and size (**a**–**h**) and SEM images (**i**–**l**) of 3D-printed polycaprolactone/gelatin scaffolds supplemented with different concentrations of bacterial cellulose and hydroxyapatite for bone tissue engineering. Reprinted from [83] under a Creative Commons Attribution License 4.0 (CC BY), “CC BY 4.0 Deed|Attribution 4.0 International|Creative Commons (accessed on 30 May 2024)”.

SEM also allowed observations for tuning of fine characteristics, like pore shapes from round to square and sizes between 301 and 314 µm in 3D-printed polycaprolactone/gelatin scaffolds, via the addition of different concentrations of bacterial cellulose and hydroxyapatite in the hydrogel composition, to better meet the required mechanical properties and to facilitate the interaction with cells for bone tissue engineering applications (Figure 9) [83].

**Figure 10:**
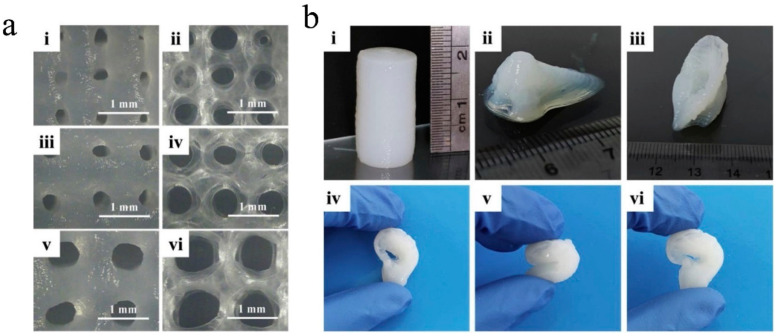
(**a**) Images of bacterial cellulose/alginate and laponite nanoclay printed hydrogel at a line spacing between 0.8 and 1.2 mm (i–vi), before and after freeze-drying. (**b**) Cylinder (i), nose (ii), and ear (iii–vi) structures maintaining high-fidelity shape and stability, reprinted from [76], Copyright (2020), with permission from Elsevier, under a Creative Commons Attribution License 4.0 (CC BY), “CC BY 4.0 Deed|Attribution 4.0 International|Creative Commons (accessed on 30 May 2024)”.

Structural details of bacterial nanocellulose/alginate 3D-printed porous scaffolds before and after oxidization with (2,2,6,6-tetramethylpiperidin-1-yl)oxyl (TEMPO) and laponite nanoclay incorporation were provided with a high degree of precision via field emission SEM [76]. The team also reported that this composition allowed the preparation of porous 3D-printed scaffolds with different pore sizes, which varied according to the used working parameters, such as line spacing, but were also suitable for multiple-layered materials printed as complex nose- and ear-like structures with high-fidelity shape and stability for long-term preservation (Figure 10).

High-resolution topographical images obtained via AFM allowed the calculation of the fiber-length-to-diameter ratio of bacterial cellulose nanofibers, highlighting the positive interaction with maleic acid to decrease their diameter and to further use combinations with gelatin for high-precision stable 3D-printed osteogenic constructs [81].

With regard to mechanical property characterization, various bacterial nanocellulose-based bioprinted scaffolds were tested, and the effect of the composition and crosslinking was assessed. Thus, the influence of component concentration, including bacterial nanocellulose concentration, on the mechanical properties of composite hydrogels with gelatin methacryloyl, was analyzed to determine the optimal values and mixing ratios for obtaining reinforced 3D-printed cartilage [69].

Confocal laser scanning microscopy observations of living materials with self-healing and self-regenerating capabilities manufactured via the 3D printing of xanthan gel incorporating *Gluconacetobacter xylinus* revealed the morphology of a heterogeneous cellulose network produced by bacteria in situ after incubation in culture medium [84].

## 4. Conclusions and Future Development

Ongoing research efforts are focused on expanding the utility of bacterial cellulose in more sophisticated biofabrication applications, including vascularized tissue constructs, organoids, and bioartificial organs. Future studies might explore novel approaches for enhancing the functionalization of bacterial cellulose hydrogels with bioactive molecules, growth factors, and cell-laden bioinks to create biomimetic tissues with improved physiological relevance and therapeutic efficacy.

Looking ahead to the future, bacterial cellulose is poised to emerge as a key player in revolutionizing the field of biomedical 3D printing. With ongoing advancements in research and technology, the potential applications of bacterial cellulose are expanding rapidly, particularly in the areas of tissue engineering, regenerative medicine, and personalized healthcare. Future developments may involve the integration of bacterial cellulose with bioactive molecules, stem cells, or growth factors to create functionalized constructs that closely mimic native tissue structures and promote enhanced tissue regeneration. The further optimization of 3D printing techniques utilizing bacterial cellulose-based materials is anticipated to lead to the fabrication of patient-specific implants, organ-on-a-chip devices, and advanced drug delivery systems tailored to individual patient needs. The continued exploration of bacterial cellulose in the realm of 3D printing holds significant promise for addressing crucial healthcare challenges and driving forward the development of cutting-edge therapies and interventions that have the potential to positively impact patients on a global scale.

## Figures and Tables

**Table 1:** Advantageous characteristics of bacterial cellulose vs. plant-derived cellulose that are useful for obtaining hydrogels and 3D-printed scaffolds for tissue engineering.

	Bacterial Cellulose	Plant-Derived Cellulose
Composition	High purity	Contains impurities (pectin, hemicellulose, lignin)
Structure	Network of nanofibrils	Complicated ribbon structure
	High porosity with uniform distribution of pore size	Low porosity of native cellulose
Physicochemical characteristics	Slow degradation in physiological medium	Difficult to degrade in physiological medium
	High crystallinity (up to 85%)	Low crystallinity (40–60%)
	Optimal tensile strength and flexibility	High tensile strength and rigidity
	Great thermal stability due to crystallinity and purity	Good thermal stability
Biological properties	No cytotoxicity, no inflammatory response	Low or no cytotoxicity
	Good cell adhesion, in particular in solid form	Good cell adhesion
	No native antimicrobial activity, but has an optimal structure as a scaffold for antimicrobial agents	No native antimicrobial activity

**Table 2:** Applications of bacterial-cellulose-based materials as inks and 3D-printed scaffolds for tissue engineering. BC—bacterial cellulose.

Hydrogel Formulation	Printing Type	Physicochemical Characteristics	Biological Properties	Reference
Silk and gelatin 1:2 (*w*/*w*) crosslinked with glycerol 5:1 (*w*/*w*) and BC add at 0.35–1.40 wt%, formulated as 3D scaffolds for human meniscus	Integration of 3D printing technology with lyophilization on 4th-generation 3D Bioplotter™ (EnvisionTEC GmbH, Gladbeck, Germany)	Maintaining shape when printing soft tissue models; viscosity influenced by the levels of silk fibroin and gelatin rather than proportions of BC; higher shape fidelity by adding BC nanofibers; BC is increased the mechanical properties and match the mechanical requirement of native soft tissues	In vitro and in vivo tests revealed that the printed scaffolds with hierarchical pores were able to facilitate cell infiltration and tissue ingrowth; an in vitro test demonstrated that the composite scaffolds exhibited outstanding biocompatibility	[74]
Alginate (4 wt%)/BC/Copper (2.5 wt%) hydrogel composites formulated as novel antimicrobial biomaterials	3D printing technology on 4th generation 3D Bioplotter (EnvisionTEC, Germany).	Incorporation of BC nanofibrils at concentrations above 50% had a notable impact on the rheological characteristics of alginate solutions; BC/alginate scaffolds (70/30 wt%) with ionic crosslinking possessed long-term dimensional stability; incorporating BC nanofibrils further enhanced the printability of alginate inks	Inclusion of copper conferred targeted antimicrobial activity against *E. coli* and *S. aureus* strains on materials	[75]
2,2,6,6-tetramethylpiperidinyl-1-oxyl (TEMPO)-oxidized bacterial cellulose, sodium alginate and laponite nanoclay, formulated as a biomedical device for drug release and tissue engineering	standard 3D printer (CoLiDo DIY, Zhuhai, China) modified	Mechanical properties were similar to those of the pericellular matrix of human cartilage (17−200 kPa); the structure was nanofiber-reinforced biomimetic	Long-term sustained release of BSA; structural stability in PBS for over 14 days and the ability to promote cell growth; beneficial to tissue growth and replacement	[76]
Regenerated silk fibroin and TEMPO-oxidized bacterial cellulose nanofibrils (2/1, 1/1, 1/2, and 0/1) with HRP and H_2_O_2_ solution 5% (*v*/*v*), designed to function as scaffolds in lung tissue engineering	BioScaffolder Printer 4.2 (GeSim, Radeberg, Germany) with a 410 lm nozzle	Composite inks displayed precise dynamic yield stress values (63–593 Pa); addition of oxidized BC (7 wt%) enhanced the viscosity of the inks, printability, and further improved the shape accuracy of the scaffolds; 3D construct with ten printed layers and porous structure after swelling in deionized water to equilibrium; immersion in alcohol improved the mechanical properties	cells displayed an anisotropic topography and arranged themselves along the longitudinal axis in the direction of the BC nanofibrils.	[77]
TEMPO-oxidized BC and maleic acid with gelatin addition, formulated as scaffolds for bone regeneration	modified 3D printer (Bio-Architect, Regenovo, Hangzhou, China)	Good printability, with the printed models showcasing smooth lines, clear grid spacing, and consistent lines; printable ear models and bone models without collapsing; dispersions had individual nanofibers with rod- and thin-strip-shaped morphologies that efficiently supported the printed scaffold’s internal framework pores	In vitro testing showed elevated ALP activity, osteoblast cultures with high cell survival, the expression of osteogenic marker genes, and mineralized nodule formation; in vivo enhancement of newly formed bone and trabecular thickness in the calvaria of rats, promoting bone regeneration	[2]
Maleic acid-treated BC combined with gelatin to create scaffolds for bone tissue regeneration	3D bioprinter (Bio-Architect, Regenovo, China).	60% maleic acid treatment maintained surface wettability of scaffolds and had the best crystallinity index and mechanical properties; enhanced rheological properties, compression modulus, and use as bioink for 3D bioprinting	Excellent in vitro biocompatibility with MC3T3-E1 cells, notable capability for alkaline phosphatase expression, formation of mineralized nodules, and expression of osteogenic-related genes throughout the differentiation process	[78]
Gelatin methacryloyl/hyaluronic acid methacryloyl/BC composite, formulated as 3D-bioprinted photocurable hydrogel for nasal cartilage repair	3D-Bioplotter (EnvisionTEC, Germany)	increased BC concentration (0.75% *w/v*); the pore size decreased to 41.26 µm as a result of hydrogen bond formation; enhanced mechanical properties of hydrogels ink viscosity, shear stress, storage, and loss modulus with interactions between polymer network molecules; high water absorption, rapid rehydration ability, degradation resistance, and improved printing fidelity	In vitro cell proliferation of ATDC5 cells and scaffold structure integrity with the increase in culture days; promotion of cell viability in live/dead assay and DAPI staining	[79]
BC hydrogel matrix (bath) for deposition of silicone elastomer (polydimethylsiloxane) inks, air-dried, formulated as paper-like membrane for drug screening in economical tissue models based on human cells	Depositing blended ink through extrusion into baths of BC hydrogels using a 3D bioprinter Allevi I (Allevi Inc., Philadelphia, PA, USA)	Viscosity of BC suspensions increased with concentration; high storage modulus for use as support material; BC matrices increased stability of printed microfibers	Enabled the inward movement of individual suspended cells during the initial seeding phase; supported the subsequent migration and growth of cells within the gaps among the BC nanofibrils; allowed the creation of a fully three-dimensional tissue model for drug screening; has a suitable in vitro environment that promotes cell interactions in models of three-dimensional tissues; injected cisplatin in endothelialized microchannels exerted dose-dependent toxicity	[23]
Encapsulated microalgae in alginate hydrogel matrix (2.5 w/v%) on a dried BC membrane, formulated as bioprinted living materials	Bioprinting with an adapted DIY bioprinter (CoLiDo DIY, China)	Microalgal bioprints on BC had good mechanical properties and maintained original shapes upon unfolding, untwisting, and uncrushing; patterned microalgae in the bioprints might be renewed to produce fresh batches of bio-inks	Bioprinted microalgal cells exhibited high viability over at least 1 month; cell density increased in bioprints throughout the 28-day incubation period; were not degraded over time by the microalgae in the bioprints; bioprints adhered to a new surface of BC remain connected to it	[71]
Cellulose nanofibrils/BC hydrogel ink containing active bacteria and PTFE microparticles with an inert matrix, formulated as a form of vascular tissue engineering	custom-built 3D printer	High storage modulus; enhanced mechanical strength through ionic crosslinking; controlled printability; dimensional stability	Improved biocompatibility through collagen immobilization for novel biomedical materials	[80]
BC nanofibrils functionalized with 3-(trimethoxysilyl)propyl methacrylate (TMSPM) formulated as highly elastic 3D-reinforced gels	3D printer 3D BioScaffolder BS3.2 (GeSIM, Germany)	Preservation of nanofibrous morphology of cellulose after modification; superior mechanical properties with gels with a high elastic modulus and high strength; formation of flexible network due to chemical crosslinks; improved stability due to reduced agglomeration, resulting in smoother surface printing; improved stability of printed scaffolds	-	[61]
TEMPO-oxidized BC nanofibrils for controlled delivery of mammalian cells	Pneumatic-based direct ink writing printer SHOTmini 200Sx (Musashi Engineering, Inc., Tokyo, Japan)	Breaking down oxidized cellulose hydrogels using enzymes; printing of scaffolds with tunable mechanical strength; control of degradation profiles using geometry in scaffolds with different infill densities; tuning of hydrogel biodegradation without altering material chemistry	Embryonic stem cell encapsulation and pluripotency preservation; 3D bioprinting of liver-mimetic constructs; growth of stem cells with delayed differentiation; gene expression in response to cell encapsulation in cellulose hydrogels	[81]

## Data Availability

Not applicable.

## References

[1] Fedotova V.S., Sokolova M.P., Vorobiov V.K., Sivtsov E.V., Lukasheva N.V., Smirnov M.A. (2023). Water influence on the physico-chemical properties and 3D printability of choline acrylate—Bacterial cellulose inks. Polymers.

[2] Wang X., Tang S., Chai S., Wang P., Qin J., Pei W., Bian H., Jiang Q., Huang C. (2021). Preparing printable bacterial cellulose based gelatin gel to promote in vivo bone regeneration. Carbohydr. Polym..

[3] Wahid F., Zhong C., Binod P., Raveendran S., Pandey A. (2021). Production and applications of bacterial cellulose. Biomass, Biofuels, Biochemicals.

[4] De Amorim J.D.P., da Silva Junior C.J.G., de Medeiros A.D.M., do Nascimento H.A., Sarubbo M., de Medeiros T.P.M., Costa A.F.d.S., Sarubbo L.A. (2022). Bacterial cellulose as a versatile biomaterial for wound dressing application. Molecules.

[5] Raut M.P., Asare E., Syed Mohamed S.M.D., Amadi E.N., Roy I. (2023). Bacterial cellulose-based blends and composites: Versatile biomaterials for tissue engineering applications. Int. J. Mol. Sci..

[6] Davis S., Roldo M., Blunn G., Tozzi G., Roncada T. (2021). Influence of the mechanical environment on the regeneration of osteochondral defects. Front. Bioeng. Biotechnol..

[7] Ghilan A., Nicu R., Ciolacu D.E., Ciolacu F. (2023). Insight into the latest medical applications of nanocellulose. Materials.

[8] Magni A., Agostoni P., Bonezzi C., Massazza G., Mene P., Savarino V., Fornasari D. (2021). Management of osteoarthritis: Expert opinion on NSAIDs. Pain Ther..

[9] Finny A.S., Popoola O., Andreescu S. (2021). 3D-printable nanocellulose-based functional materials: Fundamentals and applications. Nanomaterials.

[10] Choi S.M., Rao K.M., Zo S.M., Shin E.J., Han S.S. (2022). Bacterial cellulose and its applications. Polymers.

[11] Jankau J., Błażyńska-Spychalska A., Kubiak K., Jędrzejczak-Krzepkowska M., Pankiewicz T., Ludwicka K., Dettlaff A., Pęksa R. (2022). Bacterial cellulose properties fulfilling requirements for a biomaterial of choice in reconstructive surgery and wound healing. Front. Bioeng. Biotechnol..

[12] de Olyveira G.M., Costa L.M.M., dos Santos Riccardi C., dos Santos M.L., Daltro P.B., Basmaji P., de Cerqueira Daltro G., Guastaldi A.C., Grumezescu A.M. (2016). Bacterial cellulose for advanced medical materials. Nanobiomaterials in Soft Tissue Engineering.

[13] Athukoralalage S.S., Balu R., Dutta N.K., Roy Choudhury N. (2019). 3D bioprinted nanocellulose-based hydrogels for tissue engineering applications: A brief review. Polymers.

[14] Wang F., Borjas A., Bonto A., Ursu A.V., Dupont M., Roche J., Delattre C. (2024). Exploring novel applications for hydrogels derived from modified celluloses. Polymers.

[15] Torres-Rendon J.G., Köpf M., Gehlen D., Blaeser A., Fischer H., De Laporte L., Walther A. (2016). Cellulose nanofibril hydrogel tubes as sacrificial templates for freestanding tubular cell constructs. Biomacromolecules.

[16] Oksman K., Aitomaki Y., Mathew A.P., Siqueira G., Zhou Q., Butylina S., Tanpichai S., Zhou X.J., Hooshmand S. (2016). Review of the recent developments in cellulose nanocomposite processing. Compos. Part A Appl. Sci..

[17] Berns E.J., Sur S., Pan L., Goldberger J.E., Suresh S., Zhang S., Kessler J.A., Stupp S.I. (2014). Aligned neurite outgrowth and directed cell migration in self-assembled monodomain gels. Biomaterials.

[18] Goffin A.L., Raquez J.M., Duquesne E., Siqueira G., Habibi Y., Dufresne A., Dubois P. (2011). Poly(epsilon-caprolactone) based nanocomposites reinforced by surface-grafted cellulose nanowhiskers via extrusion processing: Morphology, rheology, and thermo-mechanical properties. Polymers.

[19] Dima S.O., Panaitescu D.-M., Orban C., Ghiurea M., Doncea S.M., Fierascu R.C., Nistor C.L., Alexandrescu E., Nicolae C.A., Trică B. (2017). Bacterial nanocellulose from side-streams of Kombucha beverages production: Preparation and physical-chemical properties. Polymers.

[20] Rahman M.M., Netravali A.N. (2016). Aligned bacterial cellulose arrays as “green” nanofibers for composite materials. ACS Macro Lett..

[21] Dutta S.D., Patel D.K., Lim K.T. (2019). Functional cellulose-based hydrogels as extracellular matrices for tissue engineering. J. Biol. Eng..

[22] Suryanto H., Muhajir M., Sutrisno T.A., Zakia N., Yanuhar U. (2019). The mechanical strength and morphology of bacterial cellulose films: The effect of NaOH concentration. IOP Conf. Ser. Mater. Sci. Eng..

[23] Li H., Cheng F., Li W., Cao X., Wang Z., Wang M., Robledo-Lara J.A., Liao J., Chávez-Madero C., Hassan S. (2020). Expanding sacrificially printed microfluidic channel-embedded paper devices for construction of volumetric tissue models in vitro. Biofabrication.

[24] Naomi R., Idrus R.B.H., Fauzi M.B. (2020). Plant- vs. bacterial-derived cellulose for wound healing: A review. Int. J. Environ. Res. Public Health.

[25] Kirdponpattara S., Phisalaphong M., Kongruang S. (2017). Gelatin-bacterial cellulose composite sponges thermally cross-linked with glucose for tissue engineering applications. Carbohydr. Polym..

[26] Pahlevan M., Toivakka M., Alam P. (2018). Mechanical properties of TEMPO-oxidised bacterial cellulose-amino acid biomaterials. Eur. Polym. J..

[27] Backdahl H., Helenius G., Bodin A., Nannmark U., Johansson B.R., Risberg B., Gatenholm P. (2006). Mechanical properties of bacterial cellulose and interactions with smooth muscle cells. Biomaterials.

[28] Mangayil R.K., Rajala S.M., Pammo A.J., Sarlin E.L., Luo J., Santala V.P., Karp M.T., Tuukkanen S.R. (2017). Engineering and characterization of bacterial nanocellulose films as low cost and flexible sensor material. ACS Appl. Mater. Interfaces.

[29] Agrawal A., Hussain C.M. (2023). 3D-Printed Hydrogel for Diverse Applications: A Review. Gels.

[30] Yao J., Chen S., Chen Y., Wang B., Pei Q., Wang H. (2017). Macrofibers with High Mechanical Performance Based on Aligned Bacterial Cellulose Nanofibers. ACS Appl. Mater. Interfaces.

[31] Liu S., Yu J.M., Gan Y.C., Qiu X.Z., Gao Z.C., Wang H., Chen S.X., Xiong Y., Liu G.H., Lin S.E. (2023). Biomimetic natural biomaterials for tissue engineering and regenerative medicine: New biosynthesis methods, recent advances, and emerging applications. Mil. Med. Res..

[32] Shin H., Jo S., Mikos A.G. (2003). Biomimetic materials for tissue engineering. Biomaterials.

[33] Markstedt K., Mantas A., Tournier I., Martínez Á.H., Hägg D., Gatenholm P. (2015). 3D Bioprinting Human Chondrocytes with Nanocellulose–Alginate Bioink for Cartilage Tissue Engineering Applications. Biomacromolecules.

[34] Chen S.Q., Lopez-Sanchez P., Wang D., Mikkelsen D., Gidley M.J. (2018). Mechanical properties of bacterial cellulose synthesised by diverse strains of the genus *Komagataeibacter*. Food Hydrocoll..

[35] Starborg T., Kalson N.S., Lu Y., Mironov A., Cootes T.F., Holmes D.F., Kadler K.E. (2013). Using transmission electron microscopy and 3View to determine collagen fibril size and three-dimensional organization. Nat. Protoc..

[36] Abouzeid R.E., Khiari R., Salama A., Diab M., Beneventi D., Dufresne A. (2020). In situ mineralization of nano-hydroxyapatite on bifunctional cellulose nanofiber/polyvinyl alcohol/sodium alginate hydrogel using 3D printing. Int. J. Biol. Macromol..

[37] Mietner J.B., Willruth S., Komban R., Gimmler C., Nehmeh B., Navarro J.R.G. (2023). Polymer-modified cellulose nanofibrils cross-linked with cobalt iron oxide nanoparticles as a gel ink for 3D printing objects with magnetic and electrochemical properties. Fibers.

[38] Chen N., Luo F.Y., Yang G.W., Yao J.R., Chen X., Shao Z.Z. (2024). Production of functional materials derived from regenerated silk fibroin by utilizing 3D printing and biomimetic enzyme-induced mineralization. Chin. J. Polym. Sci..

[39] Xu W., Molino B.Z., Cheng F., Molino P.J., Yue Z., Su D., Wang X., Willför S., Xu C., Wallace G.G. (2019). On low-concentration inks formulated by nanocellulose assisted with gelatin methacrylate (GelMA) for 3D printing toward wound healing application. ACS Appl. Mater. Interfaces.

[40] Fermani M., Platania V., Kavasi R.-M., Karavasili C., Zgouro P., Fatouros D., Chatzinikolaidou M., Bouropoulos N. (2021). 3D-printed scaffolds from alginate/methyl cellulose/trimethyl chitosan/silicate glasses for bone tissue engineering. Appl. Sci..

[41] Shojaeiarani J., Rasouli R., Frampton J. (2023). Coaxial bioprinting of cellulose nanocrystal-reinforced core-sheath strands for alginate hydrogel construct fabrication. Carbohydr. Polym. Technol. Appl..

[42] Ajdary R., Huan S., Zanjanizadeh Ezazi N., Xiang W., Grande R., Santos H.A., Rojas O.J. (2019). Acetylated nanocellulose for single-component bioinks and cell proliferation on 3D-printed scaffolds. Biomacromolecules.

[43] Stanco D., Boffito M., Bogni A., Puricelli L., Barrero J., Soldati G., Ciardelli G. (2020). 3D Bioprinting of Human Adipose-Derived Stem Cells and Their Tenogenic Differentiation in Clinical-Grade Medium. Int. J. Mol. Sci..

[44] Brusentsev Y., Yang P., King A.W.T., Cheng F., Cortes Ruiz M.F., Eriksson J.E., Kilpeläinen I., Willför S., Xu C., Wågberg L. (2023). Photocross-Linkable and Shape-Memory Biomaterial Hydrogel Based on Methacrylated Cellulose Nanofibres. Biomacromolecules.

[45] Popa L., Ghica M.V., Tudoroiu E.-E., Ionescu D.-G., Dinu-Pîrvu C.-E. (2022). Bacterial Cellulose—A Remarkable Polymer as a Source for Biomaterials Tailoring. Materials.

[46] Kutova A., Stankova L., Vejvodova K., Kvitek O., Vokata B., Fajstavr D., Kolska Z., Broz A., Bacakova L., Svorcik V. (2021). Influence of drying method and argon plasma modification of bacterial nanocellulose on keratinocyte adhesion and growth. Nanomaterials.

[47] Zheng L., Li S., Luo J., Wang X. (2020). Latest advances on bacterial cellulose-based antibacterial materials as wound dressings. Front. Bioeng. Biotechnol..

[48] Kupnik K., Primožič M., Kokol V., Leitgeb M. (2020). Nanocellulose in drug delivery and antimicrobially active materials. Polymers.

[49] Berndt S., Wesarg F., Wiegand C., Kralisch D., Mueller F.A. (2013). Antimicrobial porous hybrids consisting of bacterial nanocellulose and silver nanoparticles. Cellulose.

[50] Maneerung T., Tokura S., Rujiravanit R. (2008). Impregnation of silver nanoparticles into bacterial cellulose for antimicrobial wound dressing. Carbohydr. Polym..

[51] Luan J., Wu J., Zheng Y., Song W., Wang G., Guo J., Ding X. (2012). Impregnation of silver sulfadiazine into bacterial cellulose for antimicrobial and biocompatible wound dressing. Biomed. Mater..

[52] Liu C., Yang D., Wang Y., Shi J., Jiang Z. (2012). Fabrication of antimicrobial bacterial cellulose–Ag/AgCl nanocomposite using bacteria as versatile biofactory. J. Nanopart. Res..

[53] Xiong R., Lu C., Zhang W., Zhou Z., Zhang X. (2013). Facile synthesis of tunable silver nanostructures for antibacterial application using cellulose nanocrystals. Carbohydr. Polym..

[54] Gan I., Chow W.S. (2018). Antimicrobial poly(lactic acid)/cellulose bionanocomposite for food packaging application: A review. Food Packag. Shelf Life.

[55] Jipa I.M., Stoica-Guzun A., Stroescu M. (2012). Controlled release of sorbic acid from bacterial cellulose based mono and multilayer antimicrobial films. LWT.

[56] Butchosa N., Brown C., Larsson P.T., Berglund L.A., Bulone V., Zhou Q. (2013). Nanocomposites of bacterial cellulose nanofibers and chitin nanocrystals: Fabrication, characterization and bactericidal activity. Green Chem..

[57] Oz Y.E., Erdogan Z.K., Safa N., Hames E.E. (2021). A review of functionalised bacterial cellulose for targeted biomedical fields. J. Biomat. Appl..

[58] Portal O., Clark W.A., Levinson D.J. (2009). Microbial cellulose wound dressing in the treatment of nonhealing lower extremity ulcers. Wounds.

[59] Chen J., Zhuang A., Shao H., Hua X., Zhang Y. (2017). Robust silk fibroin/bacterial cellulose nanoribbon composite scaffolds with radial lamellae and intercalation structure for bone regeneration. J. Mater. Chem. B.

[60] Aditya T., Allain J.P., Jaramillo C., Restrepo A.M. (2022). Surface Modification of Bacterial Cellulose for Biomedical Applications. Int. J. Mol. Sci..

[61] Prosvirnina A.P., Bugrov A.N., Dobrodumov A.V., Vlasova E.N., Fedotova V.S., Nikolaeva A.L., Vorobiov V.K., Sokolova M.P., Smirnov M.A. (2022). Bacterial cellulose nanofibers modification with 3-(trimethoxysilyl)propyl methacrylate as a crosslinking and reinforcing agent for 3D printable UV-curable inks. J. Mater. Sci..

[62] Smirnov M.A., Fedotova V.S., Sokolova M.P., Nikolaeva A.L., Elokhovsky V.Y., Karttunen M. (2021). Polymerizable Choline- and Imidazolium-Based Ionic Liquids Reinforced with Bacterial Cellulose for 3D-Printing. Polymers.

[63] Amin M.C.I.M., Ahmad N., Halib N., Ahmad I. (2017). Synthesis and characterization of thermo- and pH-responsive bacterial cellulose/acrylic acid hydrogels for drug delivery. Carbohydr. Polym..

[64] Sun B., Wang P., Zhang J., Lin J., Sun L., Wang X., Chen C., Sun D. (2024). In situ biosynthesis of bacterial cellulose hydrogel spheroids with tunable dimensions. J. Biores. Bioprod..

[65] Treesuppharat W., Rojanapanthu P., Siangsanoh C., Manuspiya H., Ummartyotin S. (2017). Synthesis and characterization of bacterial cellulose and gelatin-based hydrogel composites for drug-delivery systems. Biotechnol. Rep..

[66] Atykyan N., Revin V., Shutova V. (2020). Raman and FT-IR Spectroscopy investigation the cellulose structural differences from bacteria *Gluconacetobacter sucrofermentans* during the different regimes of cultivation on a molasses media. AMB Express.

[67] Khattak S., Qin X.T., Huang L.H., Xie Y.Y., Jia S.R., Zhong C. (2021). Preparation and characterization of antibacterial bacterial cellulose/chitosan hydrogels impregnated with silver sulfadiazine. Int. J. Biol. Macromol..

[68] Laromaine A., Tronser T., Pini I., Parets S., Levkin P.A., Roig A. (2018). Free-standing three-dimensional hollow bacterial cellulose structures with controlled geometry via patterned superhydrophobic–hydrophilic surfaces. Soft Matter.

[69] Zeng J., Jia L., Wang D., Chen Z., Liu W., Yang Q., Liu X., Jiang H. (2023). Bacterial nanocellulose-reinforced gelatin methacryloyl hydrogel enhances biomechanical property and glycosaminoglycan content of 3D-bioprinted cartilage. Int. J. Bioprint..

[70] Gorbaciova T., Melenevsky Y., Cohen M., Cerniglia B.W. (2018). Osteochondral lesions of the knee: Differentiating the most common entities at MRI. Radiographics.

[71] Balasubramanian S., Yu K., Meyer A.S., Karana E., Aubin-Tam M.E. (2021). Bioprinting of Regenerative Photosynthetic Living Materials. Adv. Funct. Mater..

[72] Du H., Liu W., Zhang M., Si C., Zhang X., Li B. (2019). Cellulose nanocrystals and cellulose nanofibrils based hydrogels for biomedical applications. Carbohydr. Polym..

[73] Deshpandea P., Wankara S., Mahajana S., Patilc Y., Rajwaded J., Kulkarni A. (2023). Bacterial cellulose: Natural biomaterial for medical and environmental applications. J. Nat. Fibers.

[74] Li H., Du X., Fan S., Yang G., Shao H., Li D., Cao C., Zhu Y., Zhu M., Zhang Y. (2019). Bacterial cellulose nanofibers promote stress and fidelity of 3D-printed silk based hydrogel scaffold with hierarchical pores. Carbohydr. Polym..

[75] Gutierrez E., Burdiles P.A., Quero F., Palma P., Olate-Moya F., Palza H. (2019). 3D Printing of Antimicrobial Alginate/Bacterial-Cellulose Composite Hydrogels by Incorporating Copper Nanostructures. ACS Biomater. Sci. Eng..

[76] Wei J., Wang B., Li Z., Wu Z., Zhang M., Sheng N., Liang Q., Wang H., Chen S. (2020). A 3D-printable TEMPO-oxidized bacterial cellulose/alginate hydrogel with enhanced stability via nanoclay incorporation. Carbohydr. Polym..

[77] Li H., Wei Y., Yue H., Fan S., Yao X., Ren T., Song L., Yang G., Zhang Y. (2021). 3D printed hydrogels with oxidized cellulose nanofibers and silk fibroin for the proliferation of lung epithelial stem cells. Cellulose.

[78] Wang X., Zhang Y., Luo J., Xu T., Si C., Oscanoa A.J.C., Tang D., Zhu L., Wang P., Huang C. (2023). Printability of hybridized composite from maleic acid treated bacterial cellulose with gelatin for bone tissue regeneration. Adv. Compos. Hybrid Mater..

[79] Sang S., Ma Z., Cao Y., Shen Z., Duan J., Zhang Y., Wang L., An Y., Mao X., An Y. (2023). BC enhanced photocurable hydrogel based on 3D bioprinting for nasal cartilage repair. Int. J. Polym. Mat. Polym. Biomater..

[80] Shin S., Kwak H., Shin D., Hyun J. (2019). Solid matrix-assisted printing for three-dimensional structuring of a viscoelastic medium surface. Nat. Commun..

[81] Wang X., Wu D., Liao W., Pei W., Liu Y., Gu J., Wang P., Lan K., Huang C. (2024). Constructing osteo-inductive bio-ink for 3D printing through hybridization of gelatin with maleic acid modified bacterial cellulose by regulating addition volumes of maleic acid solution. J. Biores. Bioprod..

[82] Wu Y., Wang Y., Wang F., Huang Y., He J. (2022). Preparation of 3D Printed Polylactic Acid/Bacterial Cellulose Composite Scaffold for Tissue Engineering Applications. Polymers.

[83] Cakmak A.M., Unal S., Sahin A., Oktar F.N., Sengor M., Ekren N., Gunduz O., Kalaskar D.M. (2020). 3D printed polycaprolactone/gelatin/bacterial cellulose/hydroxyapatite composite scaffold for bone tissue engineering. Polymers.

[84] Binelli M.R., Ruhs P.A., Pisaturo G., Leu S., Trachsel E., Studart A.R. (2022). Living materials made by 3D printing cellulose-producing bacteria in granular gels. Biomater. Adv..

